# Oligofructose Provides Laxation for Irregularity Associated with Low Fiber Intake

**DOI:** 10.3390/nu9121372

**Published:** 2017-12-18

**Authors:** Randal K. Buddington, Cavita Kapadia, Franka Neumer, Stephan Theis

**Affiliations:** 1 School of Health Studies, University of Memphis, Memphis, TN 38152, USA; vita583@yahoo.com; 2 BENEO-Institute, c/o BENEO GmbH, Wormser Str. 11, 67283 Obrigheim, Germany; Franka.Neumer@beneo.com (F.N.); Stephan.Theis@beneo.com (S.T.)

**Keywords:** constipation, fermentable, fiber, fructan, clinical trial, inulin, irregularity, laxation, supplement

## Abstract

Inadequate dietary fiber intake contributes to the prevalent irregularity and constipation in Western countries. Although eating adequate amounts of fibers from fiber-rich foods, foods with added fibers and dietary fiber supplements is considered the first option for improving laxation, the efficacy can vary among types of fibers. The present study is a randomized control trial that included healthy adult participants with ≤3 bowel movements/week and a habitual low dietary fiber intake in a parallel design to evaluate the benefits for laxation by supplementing the daily diet with oligofructose (Orafti^®^ P95; OF), a fermentable source of fiber and established prebiotic (*n* = 49); maltodextrin was the placebo (*n* = 48). After a run-in phase, OF was initially provided at 5 g/day, then increased to 10 and 15 g/day with four weeks for each phase. Stool frequency (bowel movements per week) for the OF and maltodextrin (MD) groups were initially similar (3.98 ± 1.49 vs. 4.06 ± 1.48), did not change for the placebo group, but increased for the OF group with the difference significant at 15 g/day (*p* = 0.023). Stool consistency was similar and remained unchanged at all doses for both groups. Gastrointestinal sensations were low for both groups. Laxation benefits were especially pronounced for participants with >13 g/day habitual dietary fiber intake, with significant laxation at 10 g and 15 g OF/day (*p* = 0.04 and *p* = 0.004, respectively) A daily supplement with a short-chain inulin-type fructan derived from chicory roots, i.e., oligofructose (Orafti^®^ P95) provided a laxation effect without causing gastrointestinal (GI) distress for healthy participants with irregularity associated with low dietary fiber intake.

## 1. Introduction

Despite dietary guidelines and recommendations, dietary fiber intake by the population of the United States remains low [1], with less than 10% meeting the recommended levels for fiber intake [2,3]. Similar findings for low fiber intake among Canadian adults have been linked with constipation [4]. With the connection between fiber intake and the benefits for normal laxation, it is not surprising that an estimated 20% of the U.S. population has chronic constipation. The direct economic costs are estimated to be $2000 to $7500 per person seeking treatment, excluding drugs and other indirect costs [5].

The actual incidence of constipation in the United States could be higher, depending on the criteria used for diagnosis [6]. Although the current understanding of normal bowel patterns in the United States is limited, findings of the National Health and Nutrition Examination Survey (NHANES) study support the “3 and 3” metric of normal frequency, with a range of 3 bowel movements per day to 3 bowel movements per week as being considered as normal bowel frequency [1]. Most constipated participants have mild to moderate symptoms, may not even consider themselves to be constipated, and when concerned rely on various over-the-counter laxatives. Many of the commonly used bulking agents are not always effective or well-tolerated [7] and other products can have side effects. There is a need for safe and effective laxation products that will be well accepted and consistently consumed. Whereas the benefits of insoluble fibers for laxation have been equivocal, a recent meta-analysis indicate supplementing the diet with soluble fiber decreases the severity of chronic idiopathic constipation and reduces associated symptoms [8].

Inulin-type fructans, such as oligofructose (OF) and inulin (IN) are non-digestible carbohydrates and considered as dietary fibers. They are soluble fibers and natural food components found in many plants that are part of the normal human diet, e.g., leek, onions, wheat, garlic, chicory, and artichoke. Commercial inulin is extracted from chicory roots. Oligofructose is produced by partial hydrolysis of inulin and is a mixture of oligosaccharides composed of fructose units linked together by β(2-1)linkages [9]. Inulin-type fructans are considered as prebiotics and provide enteric and systemic benefits [10]. Fermentation of inulin-type fructans by the gut microbiome increases bacterial mass, hence fecal bulk and produces short chain fatty acids that regulate bowel motor functions and peristalsis, corresponding with improved bowel transit, stool consistency and frequency [11,12].

The present study compared the responses of healthy adult participants with self-reported low stool frequency and dietary fiber intake habitually low and inadequate intake to supplementing the diet with oligofructose (OF; Orafti^®^ P95, BENEO-Orafti S.A., Tienen, Belgium) or a placebo of maltodextrin (MD, DE 19, Agrana Staerke GmbH, Vienna, Austria). The OF was increased stepwise from 0 g/day up to a maximum of 15 g/day. Each dose of OF (5, 10 and 15 g/day) was provided for 4 weeks with stool frequency and characteristics and gastrointestinal (GI) sensations recorded throughout each phase.

## 2. Materials and Methods

**Figure 1:**
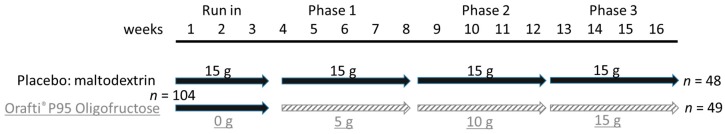
Study design.

This was a single site, randomized, controlled double blind, parallel-designed food study with a run-in and 3 treatment phases (Figure 1). The study had been approved by the University of Memphis Institutional Review Board (approval #801).

### 2.1. Participant Recruitment

The inclusion criteria for participation were being an adult (18 to 65 years), having a body mass index ≤35, 1 to 3 reported bowel movements per week, being able and willing to provide blood and stool samples, not taking fiber supplements or medications for constipation, habitually consuming a routine diet that has 50% or less of the recommended dietary fiber intake of 25 g and 38 g per day for women and men, and have access to the internet for providing logs of bowel movements, GI sensations, general health, and diet histories. Specific exclusion criteria included diabetes, existing or history of recurring GI disorders, including irritable bowel syndrome (IBS) and inflammatory bowel disease (IBD), antibiotic use within 4 weeks of starting the study, pregnancy or wishing to become pregnant, breastfeeding, abuse of alcohol or non-prescription drugs. A total of 104 volunteers were enrolled and 97 completed the study. The study population was 80% women (Table 1), corresponding with 4-fold more women than men suffering from irregularity [13].

### 2.2. Participant Assessments

During a screening visit the expectations and requirements as a participant were explained. Each potential participant’s gender, age, race, general health status, diet, and lifestyle habits were assessed. The interview included a review of relevant medical history, and particularly GI problems, chronic medications, and use of laxatives or fiber supplements in the preceding 4 weeks. If admitted into the study, informed consent was obtained, and the participant’s body weight and height were recorded and a blood sample was taken for a metabolic panel with lipids.

### 2.3. Study Protocol 

The study started with a 3 week run-in phase during which all of the participants consumed the placebo (3 sachets per day with each sachet containing 5 g of MD). Maltodextrin is a completely digestible carbohydrate, with no known effects on bowel motor function and has been used as the placebo in previous studies [12]. The participants were instructed to consume the 3 sachets (dissolved in a drink) with the main meals, and provide daily records of stool frequency and consistency and GI sensations, which were used for baseline information. At conclusion of the run-in phase the participants provided a 3-day diet history (2 weekdays, 1 weekend day) and a stool sample. The next phase started immediately and included three consecutive 4-week periods. The participants assigned to the placebo group consumed until the end of the study three sachets per day that contained 5 g of MD. Participants in the treatment group were provided dosages of OF (Orafti^®^ P95 Oligofructose) that increased for each 4-week period. For the first 4-week treatment phase, the 3 sachets consumed per day provided a combined dose of 5 g/day of OF. To have the same volume and weight as the placebo sachets, each treatment sachet contained 1.67 g of OF + 3.33 g of MD. The sachets for the next 4-week phase provided a combined quantity of 10 g/day of OF with 3.33 g of OF + 1.67 g of MD in each sachet. The sachets for the final 4-week phase provided a total of 15 g/day of OF for 4 weeks, with each sachet having 5 g of OF. Throughout the run-in and subsequent phases the participants were asked to not change their normal diet and lifestyle activities, and maintain consistent caffeine consumption. The participants provided 3-day diet histories and stool samples at the conclusion of each of the three treatment phases. All study products were packaged in identical packs (sachets) and provided in a double-blinded manner by Beneo, without revealing the code to the investigators or participants.

### 2.4. Assessments

The participants logged-in each day to a secure website to report if they had a bowel movement, any GI sensations, and if they had any health issues that might impact the response to the supplement. Stool frequency (primary efficacy variable) was the number of bowel movements per day. Stool consistency was based on the 7-point Bristol Stool Form Scale [14], with values of 3–4 considered as a normal stool consistency. The occurrence and intensity of five GI sensations that have been associated with fiber supplements (noise, pain, pressure, bloating, gas) were measured daily using a 0–10 scale. The GI sensation data for the OF and MD groups were compared for the last week of each of the four phases.

The 3 day diet histories included 2 weekdays and 1 day of the weekend (Thursday to Saturday) and were analyzed to evaluate dietary fiber intake at baseline and at the conclusion of the three treatment periods.

The stool samples at the end of each phase were collected in plastic bags, placed in insulated bags with freezer blocks, and delivered to the lab within an hour of defecation. The samples were used to measure fecal dry matter content and short-chain fatty acids (SCFA) using ion chromatography [15].

The participants also provided two fasting venous blood samples. The first was collected at the end of the run-in phase and the second at conclusion of the study. The blood samples were used for a routine metabolic diagnostic panel with lipids to obtain insights about the general health of each participant.

The participants were asked to record any adverse events during the course of the study. Adverse events were considered as events that were not expected and were not consistent with information provided in the consent form.

### 2.5. Data Analysis

Figures and tables provide means and standard deviation (SD). Normality of the data was evaluated by the Shapiro–Wilk test. Group comparisons for data that were not normally distributed were made using the Wilcoxon signed-rank test. Otherwise, comparisons between OF and MD groups were made using the Mann–Whitney-U-test. The analyses were made using the SPSS Statistics 23 software (IBM Corporation, Armonk, NY, USA) with a difference considered to be significant if *p* < 0.05.

## 3. Results

Of the 104 participants who started the study, six stopped online reporting and were removed from the study and one individual in the MD (placebo) group reported an adverse event and asked to be removed.

Antibiotic use was reported by one OF participant during the last week of the third and final four-week phase of the study. Therefore, data for this participant for the number of bowel movements, stool consistency, and GI sensations that were used for the analysis were from the week before the last week when antibiotics were taken. Another OF participant did not report GI sensations for the final week of the run-in period and values for the second week were used for the baseline.

Incomplete diet histories (two days instead of three) were provided by eight participants for one of the treatment phases. This corresponds with 2% of the diet records not being complete. For these individuals, the data reflected the average of the two reported days. One participant did not submit a diet history for one period and was excluded from the analysis for that time point.

Stool consistency was rated only when a bowel movement occurred during the week. The lack of a bowel movement in a week was reported by two of the OF and two of the MD participants. This resulted in stool consistency sample sizes of 47 and 46 for the OF and MD groups, respectively.

### 3.1. Demographics

The demographics of the 97 participants that completed the study are presented in Table 1. There were no differences between the OF and MD groups for any of the measured characteristics.

### 3.2. Dietary Fiber Intake

**Figure 2:**
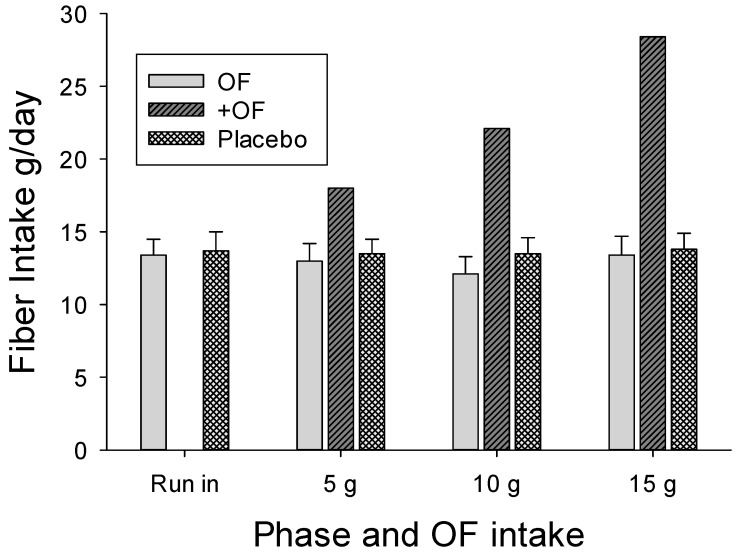
Bars showing mean habitual dietary fiber intakes of groups (with and without supplemental oligofructose (OF)) at different treatment phases. OF group: Habitual dietary fiber intake of OF group without taking into account supplemental OF. +OF group: Habitual fiber of OF group with the supplemental OF. Maltodextrin (MD) group: Placebo group.

The participant selection process was effective with the diets reported by the participants providing inadequate fiber intake throughout the study, with the average less than 14 g/day during any intake period for both groups (Figure 2). Even with the 15 g OF supplement, only 27/41 (65%) of the women in the OF group attained the recommended fiber intake of 25 g/day, but none of the men in the OF group met the recommended 38 g/day.

### 3.3. Energy Intake

Energy intake (kcal/day) was lower for the OF participants compared to the MD group for all phases of the study, and decreased as the dose of OF increased. The decline in reported energy intake was significant at 15 g/day, compared to baseline (*p* < 0.05). Reported energy intake by the placebo group was lower in the second four-week phase compared to baseline (*p* < 0.01) and the first intervention phase (*p* = 0.01), but was similar to energy intake in the final four-week phase. 

### 3.4. Stool Frequency

**Figure 3:**
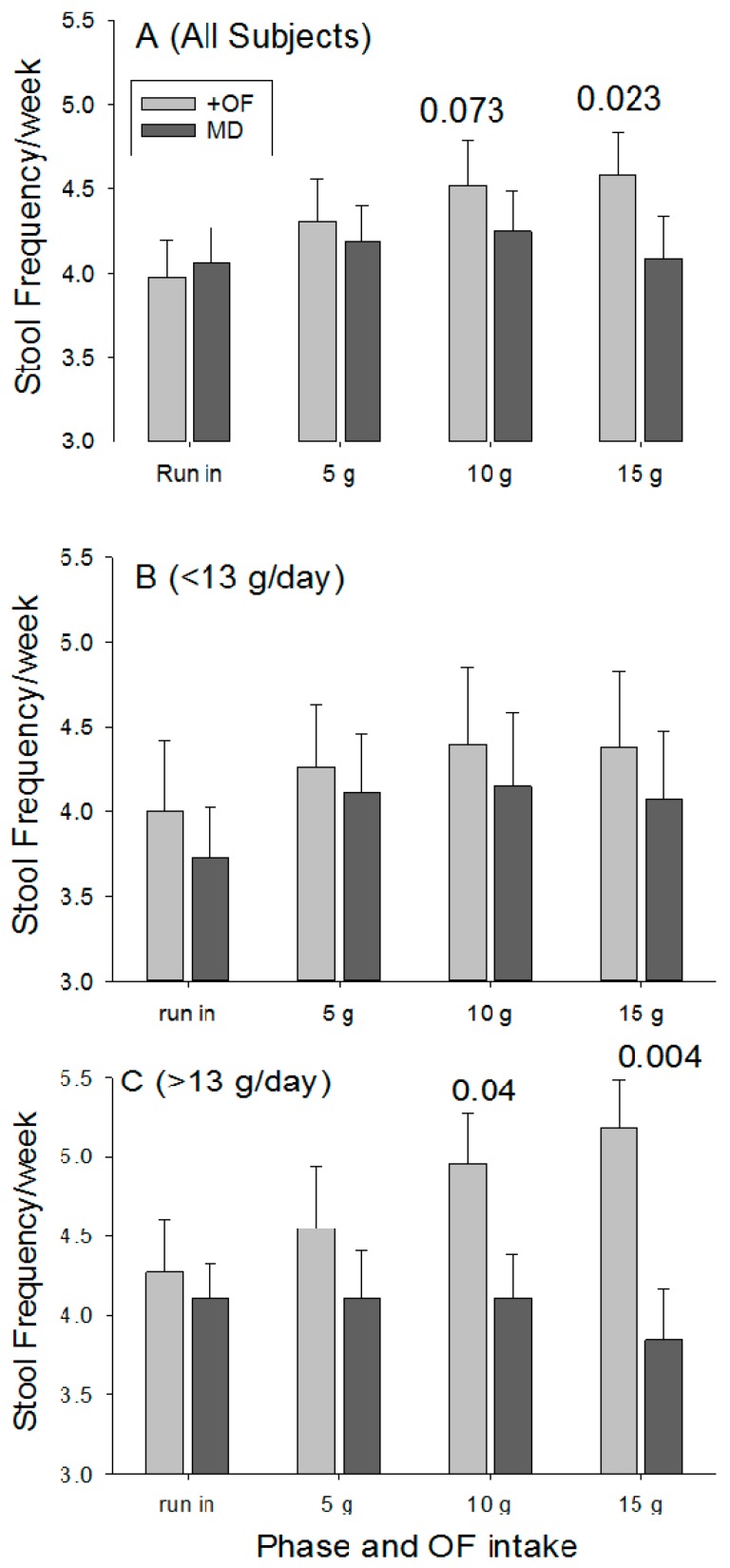
Stool frequency (number of bowel movements/week) of all study participants (**A**; +OF = 49; MD = 48) and differentiated by levels of habitual dietary fiber intake that were <13 g/day (**B**; +OF = 27; MD = 23) and >13 g/day, (**C**; +OF = 22; MD = 25). The *p*-values above the bars reflect differences with the baseline value during the run in phase. Bars without values are not different from baseline values. +OF group provided supplemental OF in addition to habitual dietary fiber intake. MD is the Placebo group.

During the run-in period the number of bowel movements per week were similar for the OF and MD (3.98 ± 1.49 vs. 4.06 ± 1.48; *p* = 0.79). Stool frequency increased as the dose of OF increased (Figure 3A), with the difference significant at 15 g/day (*p* = 0.023). Stool frequency did not change for the placebo group. 

Although a significant laxation effect was detected for the entire participant pool, restricted comparisons were made between the two groups using data for individuals that consumed <13 g of fiber per day and for those who consumed >13 g per day. This cut-off was selected based on the average fiber intake for the entire population. Providing 15 g of OF to those consuming <13 g of fiber per day (average of 9.1 ± 0.3 g/day; *n* = 27) resulted in a non-significant increase in stool frequency (Figure 3B) when compared with the run in period (*p* = 0.47) and with the placebo (*p* = 0.060). In contrast, individuals in the OF group who consumed >13 g of fiber per day (average of 17.6 ± 0.8; *n* = 22) realized increasing stool frequency as the dose increased (Figure 3C), and at 15 g/day OF there were significant differences for comparisons with the run-in period (*p* = 0.04) and with placebo group (*p* = 0.004). However, when comparisons were restricted to individuals with dietary fiber intake >20 g per, the OF participants day did not realize a significant increase in stool frequency relative to baseline (+24% ± 17%; *p* = 0.44) and did not differ from control participants (5.6 ± 0.7 vs. 5.2 + 1.8; *p* = 0.73). 

### 3.5. Stool Consistency

Bristol stool scale values during the run-in phase averaged 3.71 and 3.55 for the OF and MD groups, respectively, with both within the normal range, but consistent with irregularity. Stool consistency remained unchanged at all doses without differences between the OF and MD groups.

### 3.6. Gastrointestinal Sensations

The participants reported very low values for all five gastrointestinal sensations (Table 2). Compared to the run-in period, values for “noise”, “pressure”, and “pain” significantly decreased for OF participants, in particular when the higher doses were taken.

### 3.7. Blood Values

All values at baseline and after intervention were within the normal range and can be considered as typical of the general U.S. population (Supplemental Table S1). Gender influences were detected for baseline values (e.g., high density lipoprotein) and the responses to OF. Notably, among female participants, OF was associated with a slightly lower gamma glutamyltranspeptidase and creatinine (*p* < 0.01 each) and slightly higher HDL cholesterol (*p* < 0.01). 

### 3.8. Fecal Short Chain Fatty Acid Profiles

The concentrations of individual SCFA in the stool, whether expressed as absolute values (nmol/mg) or as percentages of total SCFA concentrations did not differ between groups at conclusion of any period. Nor did concentrations and percentages differ within a group between the run in phase to completion of the third phase (present study). This supports the notion of the overall limited informative value of fecal SCFA level due to their almost complete large intestinal absorption.

## 4. Discussion

Increasing fiber intake has the potential to reduce irregularity among individuals with a diet low in fiber [16]. However, there is debate surrounding the most effective types of fiber supplements for laxation and the amounts needed. Various forms of insoluble, bulk forming fiber (e.g., cellulose) can potentially complicate constipation. The moderate evidence for the efficacy of psyllium [17] is attributed to the soluble, gel forming component that is poorly fermented [18]. The contention that soluble, fermentable fibers do not provide laxation [19] is in contrast to several former studies and was also refuted by a recent clinical trial using chicory-derived inulin with an average degree of polymerization > 10 and participants with a dietary fiber intake of ~22 g/day [20]. The goal of this study was to determine if a shorter chain inulin-type fructan derived from chicory roots, i.e., oligofructose (OF) would provide laxation for individuals with a low fiber diet who are irregular.

The average fiber intake of the study population (13 g/day) is less than the U.S. average of ~17 g/day [2] and therefore clearly below the U.S. dietary fiber intake recommendations of 38 g/day for men and 25 g/day for women. The average fiber intake was also below those reported for populations used for the majority of previous studies evaluating the laxation responses to fructans (reviewed by [20]). Furthermore, due to changes in eating habits during recent decades it is unlikely that the majority of participants consumed the referred ~5 g of fructans in the diet of Americans during 1994 and 1996 [21]. Yet, reported stool frequency for the majority of the participants during the run-in period and throughout the study period was greater than two per week. Only two of the placebo and one of the OF participants reported stool frequencies that averaged less than two per week throughout the study and those participants had dietary fiber intakes of 9, 12, and 8 g/day, respectively. Some patients with idiopathic constipation still produce stools with low or even no-fiber diets [22]. None of the participants in either group with fiber intakes >13 g per day had a stool frequency of less than two times per week.

Our finding that the level of dietary fiber can influence the laxation response to a supplement of fructans is novel. Notably, individuals with low habitual fiber intake (an average of <13 g per day for some of the present participants) may require supplementation with more than 15 g of additional fiber per day to achieve a threshold level of fiber that will effectively stimulate laxation. The present study indicates providing 10 and 15 g of additional fiber in the form of OF is particularly effective and already sufficient for individuals with habitual intake of dietary fiber of >13 g of dietary fiber. Moreover, even at 15 g per day, the gut sensations, including bloating, experienced by the participants taking the OF supplement were minimal and did not differ from those taking the placebo. This contrasts with claims of increased bloating sensations that have been observed with fructan supplementation in some constipated participants [17]. A gradual increase in OF intake level like used for the current study would allow for the resident bacteria to adapt, reducing the likelihood of excess production of gas and eliciting additional sensations that can occur with a sudden increase in fermentable fiber.

Although this study sought participants with ≤3 bowel movements/week due to diets low in fiber, perhaps fortuitously, several individuals in each group had diets relatively high in fiber (>20 g/day; OF = 7 placebo = 5). Instead of experiencing an even more pronounced laxation response, stool frequency at 15 g of OF did not increase significantly relative to the run-in period (5.6 ± 0.7 vs. 4.9 ± 0.6; *p* = 0.44) and did not differ from that of participants assigned to the placebo (*p* = 0.73). It is possible the irregularity reported by some of these participants, despite higher fiber intake, may have underlying causes that are secondary to fiber intake. This aligns with evidence that patients with constipation caused by anorectal or colonic pathophysiology may not respond to increased dietary fiber, and other interventions are required for laxation [23].

Another consideration is the present study used OF with a lower degree of polymerization, whereas the majority of previous studies evaluating fructans used inulin or other forms of fructans with greater degrees of polymerization. This influences the distribution of fermentation along the intestine and colon. Specifically, the short chain OF is metabolized faster than inulin [24] and a greater proportion would be fermented in the ileum and proximal colon compared with the fermentation of inulin throughout the colon. The resulting OF induced increases in fecal bulking, moisture and SCFA content in the proximal colon associated with bacterial proliferation and metabolism [25] would be moderated as the digesta proceed through the remainder of the colon before being voided as stool. This is consistent with the greater fecal bulking when human subjects are provided inulin compared with OF [25] and the lack of differences between participants in the present two groups for stool consistency and SCFA concentrations. Although not recorded, it is possible the total quantity of voided stool was greater for OF participants. 

The laxation properties provided by insoluble and gel-forming fibers are direct and attributed to physical characteristics that increase fecal bulk and water content and provide viscosity associated benefits [18]. Because fructans are non-viscous and are fermented, the laxation benefits are less direct and involve other mechanisms. This includes the increased fecal bulk and water content caused by bacterial proliferation [25], improving stool consistency and facilitating voiding [26]. The SCFA produced by bacterial fermentation can act as chemical stimuli that alter tight junction permeability, hence epithelial barrier characteristics [27] and influence motility in humans [28] and animal models [29,30]. Binding of SCFA to receptors [31] appears to alter patterns of the colonic smooth muscle contraction [29] and can include a non-neural mechanism involving the release of acetylcholine influenced by the special composition and metabolic activity of the resident bacteria [32].

There are regional differences in how SCFA influence motility [28,33]. The presence of SCFA in the terminal ileum stimulates peristalsis. Recent findings reveal how gut motility provides a driving force that moves fluid into the lumen, independent of chloride secretion or osmotic gradients [34]. Therefore, the increased peristalsis will increase movement of fluid into the lumen, increasing moisture content. Interestingly, exposure of distal colon to SCFA can stimulate tonic activity that will decrease colon volume. Collectively, these responses act to move contents with greater moisture contents toward the distal colon where tonic contractions caused by the presence of SCFA would enhance defecation.

This study verifies previous reports (summarized by [20]) that soluble and fermentable fructans provide a laxation benefit for healthy human participants with a diet that doesn’t provide adequate fiber. Hence, it is not necessary for soluble fibers to form a gel to provide laxation [35]). The relationship between the laxation response to supplemental fructans and the amount of dietary fiber was previously undescribed; the influence of dietary fiber composition was not investigated and is unknown. This relationship may explain the varying degrees of laxation provided by fructans among studies that used participants with different levels of dietary fiber. The potential of the level of dietary fiber to influence laxation should be considered as a variable in designing and interpreting future studies and meta-analyses of existing data.

## Figures and Tables

**Table 1:** Baseline demographics (means and standard deviations) of the adult participants assigned to the oligofructose (OF) group or maltodextrin (MD) group (placebo).

Demographics	OF Group (*n* = 49)	MD Group (*n* = 48)	Group Differences (*p*)
Age (years)	34.2 ± 12.1	32.5 ± 10.8	0.60
Sex (% female)	80	78	0.76
Body mass index (kg/m^2^)	29.4 ± 6.3	28.7 ± 6.8	0.43
Dietary Fiber (g/day)	13.0 ± 5.1	13.6 ± 5.1	0.50
<13 g/day	9.45 ± 1.50 (*n* = 27)	9.12 ± 1.54 (*n* = 23)	0.49
13–20 g/day	15.47 ± 1.51 (*n* = 15)	15.63 ± 2.27 (*n* = 20)	0.82
>20 g/day	22.02 ± 2.77 (*n* = 7)	24.38 ± 3.23 (*n* = 5)	0.20
Bowel movements/week	3.98 ± 1.49	4.06 ± 1.48	0.79
<13 g/day	3.73 ± 1.45	4.00 ± 1.85	0.31
13–20 g/day	4.00 ± 1.46	4.05 ± 1.12	0.97
>20 g/day	4.86 ± 1.57	4.40 ± 0.89	0.62
Stool consistency	3.74 ± 1.40	3.49 ± 1.02	0.39
<13 g/day	3.92 ± 1.62	3.50 ± 1.09	0.31
13–20 g/day	3.52 ± 1.07	3.50 ± 1.02	0.98
>20 g/day	3.77 ± 1.38	3.41 ± 0.90	0.62

**Table 2:** Gut sensations (means and standard deviations) on a 0 to 10 scale reported by participants assigned to the oligofructose group + supplemental OF or the MD group (placebo) during each of the phases.

Sensation	OF Group (*n* = 49) (g/Day)				MD Group (Placebo, *n* = 48) (g/Day)			
	0	5	10	15	0	5	10	15
Noise	0.89 ^a^ ± 1.21	0.81 ^a^ ± 1.22	0.51 ^b^ ± 1.04	0.67 ^b^ ± 1.17	0.96 ± 1.95	0.83 ± 1.13	0.71 ± 1.10	0.62 ± 1.08
Pressure	0.85 ^a^ ± 1.12	0.76 ^a^ ± 1.30	0.69 ^a^ ± 0.99	0.61 ^b^ ± 0.99	0.91 ± 1.26	0.87 ± 1.21	0.61 ± 0.85	0.67 ± 0.97
Pain	0.61 ^a^ ± 0.96	0.37 ^b^ ± 0.68	0.46 ^a^ ± 0.79	0.28 ^b^ ± 0.60	0.89 ^a^ ± 1.06	0.58 ^b^ ± 0.93	0.50 ^b^ ± 0.71	0.48 ^b^ ± 0.93
Bloating	0.96 ± 1.21	0.96 ± 1.61	0.91 ± 1.44	0.90 ± 1.48	0.80 ± 1.19	0.82 ± 1.28	0.81 ± 0.94	0.78 ± 1.17
Gas	1.93 ± 2.08	1.94 ± 2.28	1.84 ± 2.17	1.87 ± 2.23	1.85 ^a,b^ ± 1.88	2.23 ^b^ ± 1.91	1.75 ^a^ ± 1.85	1.55 ^a^ ± 1.72

Values within a group with different letter superscripts are significantly different.

## Supplementary Materials

The following are available online at http://www.mdpi.com/2072-6643/9/12/1372/s1. Table S1: Blood parameters before and at conclusion of the last treatment phase for participants in the oligofructose (*n* = 45) and maltodextrin (placebo; *n* = 43) groups. Within group differences (before compared with after final treatment period) are marked with * (*p* < 0.05) or ** (*p* < 0.01). Differences between oligofructose and maltodextrin groups are marked with # (*p* < 0.05) or ## (*p* < 0.01).
