# Supplementary material for: Oligofructose Provides Laxation for Irregularity Associated with Low Fiber Intake
Source: Nutrients. 2017 Dec 18;9(12):1372. doi: 10.3390/nu9121372 (PMC5748822; doi:10.3390/nu9121372)
Supplement: Supplementary file 1 [file nutrients-09-01372-s001.pdf]

Supplemental table 1: Blood parameters before and at conclusion of the last treatment phase for participants in the oligofructose ($n$ = 45) and maltodextrin (placebo; $n$ = 43) groups. Within group differences (before compared with after final treatment period) are marked with * ($p$<0.05) or ** ($p$<0.01). Differences between oligofructose and maltodextrin groups are marked with # ($p$<0.05) or ## ($p$<0.01).

| | **Oligofructose** | | **Maltodextrin** | |
|---|---|---|---|---|
| **Blood parameter (Reference Values)** | Baseline | Treatment | Baseline | Treatment |
| Na (135-148 mEq/L) | 141.5 ± 4.0 | 142.5 ± 7.7 | 142.4 ± 4.4 | 145.5 ± 9.1 |
| K (3.5-5.5 mEq/L ) | 4.20 ± 0.35 | 4.04* ± 0.49 | 4.23 ± 0.46 | 4.22 ± 0.42 |
| Cl (98-106 mEq/L ) | 106.3 ± 5.3 | 106.5 ± 8.7 | 107.5 ± 3.9 | 110.6* ± 7.5 |
| Gluc (70-100 mg/dL ) | 84.9 ± 9.7 | 85.1 ± 12.9 | 92.5## ± 14.7 | 88.9 ± 20.7 |
| BUN (7-18 mg/dL ) | 13.08 ± 6.23 | 12.05 ± 3.82 | 13.17 ± 3.79 | 13.24 ± 4.13 |
| Creat (mg/dL) | 0.84 ± 0.71 | 0.81 ± 0.25 | 0.75 ± 0.15 | 0.82** ± 0.19 |
| Males (<1.6 mg/dL) | 0.92 ± 0.24 | 1.00 ± 0.24 | 0.85 ± 0.19 | 0.93 ± 0.25 |
| Females (<1.3 g/dL) | 0.83 ± 0.77 | 0.77** ± 0.24 | 0.72 ± 0.13 | 0.79** ± 0.15 |
| $CO_2$ (23-34 mEq/L) | 25.41 ± 4.19 | 26.77* ± 3.95 | 24.84 ± 3.07 | 26.57* ± 3.65 |
| CPK (IU/L) | 253.0 ± 437.7 | 150.9 ± 166.0 | 137.1 ± 133.5 | 169.9 ± 290.3 |
| Males(<160 IU/L) | 617.2 ± 756.2 | 256.2 ± 345.0 | 216.2# ± 254.1 | 168.1 ± 126.9 |
| Females (<130 IU/L) | 171.9 ± 285.7 | 128.3** ± 82.3 | 113.1 ± 51.9 | 170.5 ± 325.7 |
| AST (<41(IU/L ) | 27.04 ± 17.76 | 23.67 ± 9.98 | 22.65 ± 6.23 | 22.77 ± 9.72 |
| ALT (<39 (IU/L) | 18.91 ± 9.81 | 19.16 ± 9.15 | 18.49 ± 7.70 | 18.37 ± 8.10 |
| ALKP (35-123(IU/L ) | 73.69 ± 23.89 | 74.20 ± 23.49 | 73.79 ± 32.74 | 73.37 ± 36.54 |
| GGT (IU/L) | 12.20 ± 10.18 | 11.43 ± 6.82 | 13.53 ± 10.04 | 14.02 ± 11.29 |
| Males(<55 IU/L) | 20.13 ± 20.07 | 16.50 ± 9.56 | 13.10 ± 5.84 | 14.80 ± 12.36 |
| Females <36 IU/L) | 10.38 ± 5.35 | 10.11** ± 5.68 | 13.67 ± 11.08 | 13.79 ± 11.13 |
| Bili (0.2-1.2 mg/dL) | 0.74 ± 0.91 | 0.69 ± 0.47 | 0.66 ± 0.41 | 0.64 ± 0.43 |
| Alb (3.5-5.3 g/dL) | 4.21 ± 0.41 | 4.12 ± 0.48 | 4.28 ± 0.43 | 4.21 ± 0.46 |
| Ca (8.5-10.5 mg/dL) | 9.38 ± 0.52 | 9.48 ± 0.58 | 9.55 ± 0.51 | 9.76 ± 0.97 |
| Mg (1.5-2-6 mg/dL) | 2.06 ± 0.24 | 1.97** ± 0.21 | 2.05 ± 0.20 | 2.03 ± 0.30 |
| Phos (2.5-4.8 mg/dL) | 4.20 ± 0.58 | 4.16 ± 0.63 | 4.20 ± 0.67 | 4.26 ± 0.63 |
| Chol (<200 mg/dL) | 173.7 ± 37.6 | 174.6 ± 38.9 | 182.8 ± 35.0 | 184.5 ± 38.8 |
| TG (<150 mg/dL) | 90.3 ± 54.4 | 93.5 ± 63.4 | 89.9 ± 53.7 | 102.7** ± 61.5 |
| HDL (mg/dL) | 54.26 ± 14.12 | 55.63 ± 14.76 | 54.34 ± 12.76 | 54.47 ± 12.60 |
| Males (>40 mg/dL ) | 47.03 ± 16.70 | 51.35 ± 17.95 | 50.95 ± 11.30 | 45.37* ± 12.02 |
| Females >50 mg/dL) | 56.04 ± 13.07 | 56.74** ± 13.90 | 55.37 ± 13.16 | 57.22 ± 11.58 |
| LDL (<100 mg/dL) | 101.3 ± 32.3 | 100.2 ± 34.4 | 110.5 ± 30.4 | 109.5 ± 34.3 |
| Uric (2.5-7.7 mg/dL) | 4.37 ± 1.39 | 4.45 ± 1.28 | 4.54 ± 1.21 | 4.77* ± 1.27 |

Abbreviations:

Na=Sodium, K=Potassium, Cl=Chloride, Gluc=Glucose, BUN=Blood urea nitrogen, Creat=Creatinine, $CO_2$=Carbon dioxide, CPK=Creatine phosphokinase, AST=Aspartate aminotransferase, ALT=Alanine aminotransferase, ALKP=Alkaline phosphatase, GGT=Gamma glutamyltransferase, Bili=Bilirubin, Alb=Albumin, Ca=Calcium, MG=Magnesium, Phos=Phosphate, Chol=Cholesterol, TG=Triglycerides, HDL=High density lipoprotein, LDL=Low density lipoprotein, Uric=Uric acid
